# C500 variants conveying complete mucosal immunity against fatal infections of pigs with *Salmonella enterica* serovar Choleraesuis C78-1 or F18+ Shiga toxin-producing *Escherichia coli*

**DOI:** 10.3389/fmicb.2023.1210358

**Published:** 2023-09-14

**Authors:** Guoping Liu, Chunqi Li, Shengrong Liao, Aizhen Guo, Bin Wu, Huanchun Chen

**Affiliations:** ^1^College of Animal Science, Yangtze University, Jingzhou, China; ^2^Key Laboratory of Preventive Veterinary Medicine in Hubei Province, The Cooperative Innovation Center for Sustainable Pig Production, Wuhan, China; ^3^Hubei Institute of Cross Biological Health Industry Technology, Jingzhou, China; ^4^State Key Laboratory of Agricultural Microbiology, College of Veterinary Medicine, Huazhong Agricultural University, Wuhan, China

**Keywords:** *Salmonella enterica* serovar Choleraesuis C500, mucosal immunity, edema disease of swine, host, *in vivo*

## Abstract

*Salmonella enterica* serovar Choleraesuis (*S.* Choleraesuis) C500 strain is a live, attenuated vaccine strain that has been used in China for over 40 years to prevent piglet paratyphoid. However, this vaccine is limited by its toxicity and does not offer protection against diseases caused by F18+ Shiga toxin-producing *Escherichia coli* (STEC), which accounts for substantial economic losses in the swine industry. We recently generated a less toxic derivative of C500 strain with both *asd* and *crp* deletion (*S.* Choleraesuis C520) and assessed its efficacy in mice. In addition, we demonstrate that C520 is also less toxic in pigs and is effective in protecting pigs against *S.* Choleraesuis when administered orally. To develop a vaccine with a broader range of protection, we prepared a variant of C520 (*S.* Choleraesuis C522), which expresses rSF, a fusion protein comprised of the fimbriae adhesin domain *Fed*F and the Shiga toxin-producing IIe B domain antigen. For comparison, we also prepared a control vector strain (*S.* Choleraesuis C521). After oral vaccination of pigs, these strains contributed to persistent colonization of the intestinal mucosa and lymphoid tissues and elicited both cytokine expression and humoral immune responses. Furthermore, oral immunization with C522 elicited both *S.* Choleraesuis and rSF-specific immunoglobulin G (IgG) and IgA antibodies in the sera and gut mucosa, respectively. To further evaluate the feasibility and efficacy of these strains as mucosal delivery vectors via oral vaccination, we evaluated their protective efficacy against fatal infection with *S.* Choleraesuis C78-1, as well as the F18+ Shiga toxin-producing *Escherichia coli* field strain Ee, which elicits acute edema disease. C521 conferred complete protection against fatal infection with C78-1; and C522 conferred complete protection against fatal infection with both C78-1 and Ee. Our results suggest that C520, C521, and C522 are competent to provide complete mucosal immune protection against fatal infection with *S.* Choleraesuis in swine and that C522 equally qualifies as an oral vaccine vector for protection against F18+ Shiga toxin-producing *Escherichia coli*.

## 1. Introduction

F18+ Shiga toxin-producing *Escherichia coli* (*E. coli*) will cause either post-weaning diarrhea (PWD) or edema disease of swine (ED) (Niewerth et al., 2001). It is also an important causative agent of diarrhea syndrome in swine, which has emerged over the past 3 to 5 years (Nadeau et al., 2017). These two diseases are the most widespread causes of death in weaned pigs or newborn piglets and account for substantial economic losses in the swine industry (Johansen et al., 1997). Although vaccines against F4 provide good protection from the PWD caused by F4 + enterotoxigenic *Escherichia coli* (ETEC), vaccines against F18 have not shown promising results due to poor immune response and difficulty producing specific antibodies (Verdonck et al., 2007; Delisle et al., 2012; Melkebeek et al., 2013; Okello et al., 2021). Hence, there is an unmet need for research on increasing the immune response of F18 as well as providing complete protection from PWD.

ST-II e (Shiga toxin-producing two variant) and F18 fimbriae (adhesion factor) are two virulence factors that are immunogenic and are regarded as the most important components of new generation vaccines against F18+ STE C (Johansen et al., 1997; Zhao et al., 2009b; Rossi et al., 2014; Cai et al., 2019). Although the single immunogenicity of F18 fimbriae and the ST-II e is not so great, their immunogenicity is enhanced through their fusion expression (Liu et al., 2007a). ST-II e has two domains: an enzymatic subunit (A) and five copies of a cell-binding subunit (the B pentamer; 7.5 kDa × 5) (Ling et al., 1998, 2000). The B pentamer is responsible for toxin attachment to a series of glycolipids on the cell surface and has been defined as an immunodominant protective epitope (Cai and Yang, 2003). Additionally, F18 fimbriae harbor two major structural adhesin genes, *fed*A and *fed*F. Although *fed*A subunit is immunodominant as compared to *fed*F, whereas *fed*A gene is not a shared sequence and there are more variations between the antigenic variants F18ab and F18ac, the latter containing an extra proline (Snoeck et al., 2004; Facinelli et al., 2019). Another adhesion of F18 fimbriae, *fed*F gene is highly conserved among F18+ *E. coli* strains isolated in different countries, and serves as a common receptor binding site for both F18ab and F18ac, and there is no specific variation in F18ab and F18ac (Tiels et al., 2005, 2008). Furthermore, anti-*fed*F antibodies are able to inhibit F18+ *E. coli* adhesion to porcine enterocytes (O’Brien et al., 2001; Moonens et al., 2014). These facts indicate that *fed*F is another good immunogenic candidate. Further study has demonstrated rSF that fusion proteins of the fimbriae adhesin domain of *fed*F with the Shiga toxin-producing IIe B domain antigen (rSF) result in high levels of neutralizing antibody against F18+ STEC in rabbits, conferring higher immunogenicity than *fed*F or ST-IIe B subunit alone under *in vivo* conditions (Liu et al., 2007a). Therefore, rSF provides a stable foundation for the development of a novel vaccine design.

Over the last decade, recombinant attenuated *Salmonella* vaccine strains have been increasingly employed for heterologous antigen delivery (DiGiandomenico et al., 2004; Su et al., 2021). The advantage of oral delivery of these strains is their ability to activate systemic immunity including cellular immunity, humoral immunity and mucosal immunity without causing significant side effects (DiGiandomenico et al., 2004). Varied vaccine components can affect the immune responses elicited by live *Salmonella*-vectors, including expression level, location and time of antigens (Isoda et al., 2007). Diverse methods have been developed to allow well-monitored and stable delivery of antigens and augmented immunogenicity where required. This includes the selection of heterologous protective fragments and their expression under the control of suitable plasmids within the vector strains (Spreng et al., 2006). The availability of well-characterized attenuated mutants of *Salmonella* supports fine-tuning of the immune response elicited by heterologous immunogenic fragments (Spreng et al., 2006).

The C500 strain of *S.* Choleraesuis is an attenuated vaccine strain attenuated by chemical methods (Xu et al., 2006). This strain exhibits efficacy and safety and has been used to prevent and control piglet paratyphoid in China for over 40 years (Zhao et al., 2009a). It has also been developed as a potential live oral vaccine vector for heterologous antigen delivery (Zhao et al., 2008; Li et al., 2014). Although the complete genome of C500 has been characterized (Han et al., 2014), the molecular mechanism of virulence attenuation of C500 remains obscure (Ji et al., 2015). Moreover, it has residual toxicity, which limits its utility. Therefore, a more effective, more stable, and safer strain is desirable. The C500 *asd*- vaccine strain was created by the introduction of an aspartate-semialdehyde dehydrogenase (*asd*) deletion mutant, which was employed to deliver foreign antigens utilizing the *Asd* + balanced-lethal host-vector system without any antibiotic resistance gene markers (Xu et al., 2006). Zhao et al. (2009a) have developed a version of C500 based on this mutant that efficiently and consistently expresses the recombinant filamentous hemagglutinin type I domain and pertactin region 2 domain antigens (rF1P2) of *Bordetella bronchiseptica* (*Bb*). Though this variant demonstrated complete protection efficacy against lethal dose challenge via subcutaneous injection in mice, it did not exhibit satisfactory efficacy of systemic and mucosal immunity after oral administration (Zhao et al., 2009a; Fan et al., 2021). It is not known why the C500 *asd*- vaccine strain was less effective when administered orally than subcutaneous injection; however, this strain has a weakened colonizing ability, which may explain its poor immunity in mice (Xu et al., 2006; Han et al., 2014). Notably, the latter was not performed in the natural host, swine. The digestive system of pigs is different from that of mice, and the GI (gastrointestinal) tract is not only threatened by host defenses, but also more impacted by various small molecular compounds, such as glucose and other sugars. Moreover, glucose is the best carbon source for *Salmonella* and facilitates growth in culture. In the vaccine design, the Cyclic Adenosine monophosphate (cAMP)-independent cAMP receptor protein (Crp) is postulated to protect interference by glucose, which decreases synthesis of cAMP and enhances the colonizing ability and immunogenicity of the vaccine strains (Curtiss and Kelly, 1987). *Crp* deletion augments colonizing ability and immunogenicity of *Salmonella* vaccine constructs both in mice and in pigs (Curtiss et al., 2009). To maximize the colonizing ability and induction of immune responses, guarantee attenuation, prevent reversion of virulence, and eliminate potential side effects, vaccine strains typically integrate more than one advantage for genetic constructs. Therefore, C500 with both *asd* and *crp* deletion was designed and constructed as a new vaccine strain (named “C520”) (Xu et al., 2006). However, no data regarding the *in vivo* function of this engineered strain in pigs have been reported.

In this study, we evaluated the colonization, virulence and systemic immunity of C520 in pigs and determined whether this strain elicits robust immune response and confers effective protection against challenge with homologous strains via the oral administration in swine. Additionally, we constructed a C500Δ*asd*Δ*crp* strain expressing rSF (C522) and initiated a comprehensive evaluation to determine whether C522 can provide robust immunity either to STEC or to *Salmonella* itself in an ED infection model in swine. Our results demonstrate that these vaccine strains provided improved practicable vaccine candidates against STEC.

## 2. Materials and methods

### 2.1. Bacterial strains, plasmids, primers, media, and growth conditions

The bacterial strains and plasmids used in this study are listed in Table 1. The STEC Ee strain (O139, positive for ST-IIe, F18ab) is a virulent field-type strain isolated from pigs in a farm in Wuhan during an outbreak of ED (Liu et al., 2007b). The attenuated *S.* Choleraesuis vaccine strain C500 and the wild-type, virulent parental strain C78-1 were supplied by the China Institute of Veterinary Drug Control (CIVDC, Beijing, China). C500 was chosen as the parent strain for the generation of genetically modified strains. *E. coli* and *S.* Choleraesuis cultures were grown at 37°C in Luria-Bertani (Alborali et al., 2017) broth or on LB agar plates. When required, D L-α, ε-diaminopimelic acid (D L-α, ε-DAP) (Liu et al., 2007a; Gangaiah et al., 2022) was added (50 μg/ml) for the growth of *asd*- strains (Xu et al., 2006).

**TABLE 1:** Strains and plasmids used in this study.

Strain, plasmid	Relevant characteristics	Source or references
*E. coli* DH5a	supE44 ΔlacU169 (φ80 lacZΔM15) hsdR17 recA1 endA1 gyrA96 thi-1 relA1	Takara
BL21 (DE3)	F^–^ *ompT* r^–^_B_ m^–^_B_; DE3 is a λ derivative carrying *lacI* and T7 RNA polymerase genes under placUV5 control	Takara
χ7213	Thi-1 thr-1 leuB6 fhuA21 lacY1 glnV44 Δ*asd*A4 recA1 RP4 2-Tc: Mu[λpir] Kmr	Curtiss and Kelly, 1987; Curtiss et al., 2009
χ6097 *S.* Choleraesuis	F- ara Δ(pro-lac) rpsL Δ*asd*A4 Δ [zhf-2:Tn10] thi φ80 days lacZΔM15	Curtiss and Kelly, 1987; Curtiss et al., 2009
C500	A live vaccine attenuated from C78-1 by chemical methods, used to prevent piglet paratyphoid in China; serovar 6,7:C:1,5	CIVDC^a^
C78-1	Wild type, virulent strain	CIVDC^a^
C520	Δ*asd* Δ*crp* derivative of C500	This work
C521	C500Δ*asd*Δ*crp* vaccine expressing a control vector	This work
C522	C500Δ*asd*Δ*crp* vaccine expressing rSF	This work
*E. coli* Ee	Wild type, virulent strain, originally isolated from a pig suffering from edema disease of swine	Lab stock
Plasmid pBluescript SK (+)	Phagemid cloning vector, oriColE1 oriF1(+) bla lacZa	Stratagene
pRE112	oriT oriV Δ*asd* Cmr, sacB, counterselectable suicide plasmid	Curtiss and Kelly, 1987; Curtiss et al., 2009
pYA3493	*Asd* + vector; pBR322 ori; derivative β-lactamase signal sequence-based periplasmic secretion plasmid	Curtiss and Kelly, 1987; Curtiss et al., 2009
pYA-SF	1095-bp DNA encoding the ST-IIeB and *Fed*F in pYA3493	This work

^a^China institute of veterinary drug control (Beijing, China).

### 2.2. Construction of variants of the *S. enterica* serovar Choleraesuis C500 vaccine strain

The primers used for preparation of the recombinant a virulent vaccine strains are listed in Table 2. *S.* Choleraesuis C520, which has deletions in both *crp* and *asd*, was generated from the *S.* Choleraesuis C500 vaccine strain as described previously (Xu et al., 2006). Briefly, DNA was introduced into the bacteria by electroporation. A 1048 bp upstream fragment of the *crp* gene was amplified by PCR as described, with the exception that polymerization was performed at 72°C for 2.5 min from the genomic DNA of *S.* Choleraesuis C500 strain using two pairs of primers (Accession No: AE008863; N terminal, crp1, pr1 and pr2, crp2, pr3, and pr4). The amplified fragment was cloned into the *Xba*I and *Bam*HI sites of the pBluescript II SK (+) vector to construct pSK-*crp*up. Then, the 1743-bp downstream fragment of the *crp* gene was PCR-amplified using a pair of primers (pr3 and pr4) and cloned into the *Xho*I and *Kpn*I sites of pSK-*crp*up to obtain pSKΔ*crp*, which resulted in a 320-bp deletion, including the *crp* gene fragment. The 2890-bp fragment, including composed of the upstream and downstream fragments of the *crp* gene, from an *Xba*I- and *Kpn*I-digested pSKΔ*crp* plasmid was ligated to pRE112 plasmid to yield the pREΔ*crp* suicide plasmid. Transfer of the recombinant suicide plasmid to *S.* Choleraesuis C500 was accomplished by conjugation using *E. coli* χ7213 (pRE*crp*) as the plasmid donor. Strains containing single-crossover plasmid insertions (C500*crp*: pREΔ*crp*) were isolated on plates containing chloramphenicol and DAP. Loss of the suicide vector after the second recombination event between homologous regions (i.e., allelic exchange) was selected for by using a *sacB*-based sucrose sensitivity counter selection system. The presence of the 320-bp *crp* deletion in *S.* Choleraesuis C500 was confirmed by sucrose-sensitive growth on media and by PCR using a flanking *crp* primer set (pr5 + pr6). A pRE*asd* plasmid was constructed by the same method. Then C500Δ*crp* as recipient bacterium was conjugated with the *E. coli* χ7213 (pREasd) donor. The presence of the 1,408-bp *asd* deletion in *S.* Choleraesuis C520 was confirmed by the inability of the strain to grow on media without DAP and by PCR using a flanking *asd* primer set (pa5 and pa6).

**TABLE 2:** The primer sets used for the recombinant attenuated vaccine constructs.

Gene amplified	Primer names	Primer sequences (5′-3′)	Fragment length (bp)	Underline
Upstream of *crp*	pr1	TTTTCTAGAGCTGGATGAGAGTTTTGTGG	1048	*Xba*I
	pr2	TTTGGATCCCCATTCAAGAGTCGGGTCT		*Bam*HI
Downstream of *crp*	pr3	TTTCTCGAGGCTCGTCGCTTACAAGTCAC	1743	*Xho*I
	pr4	TTTGGTACCCAGTAACTGGATGGTGTATA		*Kpn*I
*crp/crp-*	pr5	GCCATTCTGACGGAATTAACGGG	1412 (*crp*^–^)	
	pr6	TCGCGTACCCATATCAACTT	1731 (wt)	
Upstream of *asd*	pr1	TTTCTAGACGCTTTGAGCACGACTAA	2112	*Xba*I
	Pr2	TTGGATCCTGCGTTAGGAAGGGAATC		*Bam*HI
Downstream of *asd*	pr3	TTGGATCCAGGGTAGCTTAATCCCAC	2069	*Xho*I
	pr4	TTGGTACCACCGAGCGTTCATTGTCA		*Kpn*I
*asd/asd-*	pr5	TTGCTTTCCAACTGCTGAGC	1803 (wt)	
	pr6	TCCTATCTGCGTCGTCCTAC	315 (*asd*-)	
ST-IIeB	pr1	TTTGAATTCAAAGGTAAAAATTGAGTT	207	*Eco*RI
	pr2	TTTGAGCTCGTTAAACTTCACCTGGGC		*Sac*I
*fedF*	pr1	TTTGAGCTCACTCTACAAGTAGAC	894	*Sac*I
	pr2	TTAAGCTTTGGTCTACTTATTACGCGATG		*Hin*dIII

To construct *S.* Choleraesuis C522, which is a derivative of C520 that expresses rSF, fragments encoding mature ST-IIe B and *fed*F were amplified from the genomic DNA of Ee *E. coli* using two pairs of primers (pB1, pB2, and pF1, pF2) that were designed according to the ST-IIeB gene sequence (GenBank accession no: AY332411) and the *fed*F gene sequence (GenBank accession no: AFZ26250) (Liu et al., 2007a). These primers contain restriction sites (*Eco*I, *Sac*I, *Sac*I, and *Hin*dIII) to allow the direct cloning of the PCR product into pYA-3493 plasmid. Two rounds of amplification were performed in a total volume of 50 μl containing 200 μM deoxynucleoside triphosphates (dATP, dCTP, dGTP, and dTTP), 1 pmol of each primer, 5 μl of dilution buffer, and 2.5 U of Taq polymerase. Thirty cycles were performed, each consisting of a denaturing step of 1 min at 94°C, an annealing step of 60 s at 55°C, and a 60 s extension step at 72°C. The 1095 bp PCR fragment of ST-IIeB and the *fed*F fragment were purified and cloned into the *Eco*RI and *Hin*dIII sites of pYA3493, resulting in pYA-SF. In-frame cloning of pYA-SF was confirmed by nucleotide sequencing. pYA-SF (encoding rSF) was electroporated into the C500Δ*asd*Δ*crp* strain (named C520), to construct the recombinant *S.* Choleraesuis C522 (pYA-SF). To prepare a control strain, pYA3493 (vector control) was electroporated into C520, resulting in C521 (pYA3493) being constructed.

### 2.3. Characterization of bacterial phenotypes

The growth curves of the strains in LB were determined, and carbohydrate fermentation or utilization assays were processed according to the manufacturer’s protocol. MH medium, sugar fermentation tube purchased from Tianhe Microbial Reagent Co., Ltd (Hangzhou, China). The group O serovar and H antigen were identified by slide agglutination with antisera supplied by the CIVDC (Beijing, China). The rSF fragment expression in the cytoplasm and culture supernatant of C522 was monitored by 12% SDS-PAGE, and immunoblot analyses were performed with the anti-rSF rabbit polyclonal antibody as previously described (Liu, 2008). Specific band intensities were further analyzed by densitometry using the Quant Studio™ 5 real-time PCR instrument (MA, USA).

### 2.4. Immunization and sampling

Duroc × Landrace × Yorkshire (DLY) Hybrid Pigs (F18R+, 28–30 days of age) were obtained from a breeding farm in Shandong Province, China to the experimental animal house of Huazhong Agricultural University. All pigs were confirmed either to be without antibody against both O antigen of *S.* Choleraesuis and rSF by their individual ELISA or to be culture-negative for both *S.* Choleraesuis and STEC by the enrichment of rectal swabs. In the process of the study, all animal experiments were approved by the Animal Ethics Committee (AEC) of the Huazhong Agricultural University, ethics number HZAUSW-2006-0005 and were performed according to the ARRIVE guidelines. When immunizing pigs (30 days of age), 2.0 × 10^9^ CFU immunization doses of vaccine strain was administered orally in accordance with the vaccine instructions for the use of the C500 strain. A total of 128 pigs were randomly assigned to four groups, as follows: group 1 (pigs 1–32) received C522; group 2 (pigs 33–64) received C521; group 3 (pigs 65–96) received C500; group 4 (pigs 97–128) was non-infected controls, respectively, via the oral route (Narita and Ishii, 2004). Twelve pigs per group, group B, group C and group D were used for the evaluation of C521 to evaluate whether or not having ability to elicit a mucosal immune response via the oral route in swine. In addition, twelve pigs in group A were monitored as well as other three groups. Clinical manifestations and rectal temperature changes were observed daily over the first 5 days and on day 14 and 21 post-vaccination. And fecal consistency score was evaluated on a continuous scale (0–4/4) as described by Fairbrother et al. (2017). Fecal consistency scores of 2, 3, and 4 were considered as indicative of mild, moderate and severe diarrhea. Pigs with rectal temperature ≥41°C are fever (Yu et al., 2012). Additionally, rectal swabs and blood samples from each pig were collected. Autopsies were conducted as presently as possible for simultaneous death or on day 0, day 1, day 14, and day 21 post-vaccination. Three grams of spleen, mesenteric lymph nodes and Peyer’s patches were immediately prepared in triplicates for cytokine determination. To determine the bacteria counts and antibody titers in the infected organs, 1 g of tissue samples from the spleen, mesenteric lymph nodes and Peyer’s patches were homogenized in 10 ml phosphate-buffered saline (PBS). Enumeration of the bacteria strains in these organs or tissues was performed by plating a dilution series of lysates on MacConkey agar after overnight incubation at 37°C. Also, 2 ml PBS were used to wash the intestinal mucosa of the part of terminal ileum and then the wash fluid was collected. Antibody titers were determined in supernatants of homogenized organs and wash fluid. The other organ samples were fixed in 10% (w/v) buffered formalin and then subjected to histopathological examination.

An additional 20 pigs in each group were selected for protection studies. After 3 weeks of inoculation, 10 pigs were challenged with 2 × 10^10^ CFU of C78-1 and another 10 pigs with 2.5 × 10^11^ CFU of Ee strain. Monitoring and sample collection from challenged pigs were performed as described above in the post-vaccination and post-challenge periods. Necropsies were performed after simultaneous death on day 21 post-infection. Organ and tissue samples were prepared as described above. Rectal swabs and organs were examined to determine whether the orally administered live vaccine candidates or challenge strain were shed in the feces or located in the organs. Isolation and identification of the vaccine candidates and challenge strains were performed according to previously described methods (Xu et al., 2006). Blood and other samples were collected and stored at −80°C until use.

### 2.5. Cytokine response in the spleens of swine

Total RNA was extracted from the spleens of the vaccinated pigs using ISOGEN (Invitrogen). Reverse transcriptase-PCR (RT-PCR) was monitored using a one-step RNA PCR kit (Takara) with three pairs of primers according to the operating instructions. Primers are listed below: IFN-Υ (5′-GTTTTTCTGGCTCTTACTGC-3′; 5′-CTTCCGCTT TCTTAGGTTAG-3′) (Chaoprasid and Dersch, 2021); TNF-α (5-ACTGCACTTCGAGGTTATCGG-3′, 5′-GGCGACGGGCTTATCTGA-3′) (Meissonnier et al., 2008); interleukin (IL)-4 (5′-GTCTGCTTACTGGCATGTACCA-3′; 5′-GCTCCATGCACGAGTTCTTTCT-3′) (Duvigneau et al., 2005); GAPDH (5′-AACGACCCCTTCATTGAC-3′; 5′-TCCACGACATACTCAGCAC-3′).

Quantitative reverse transcriptase-PCR (qRT-PCR) was performed on the 7900HT Sequence Detection System (Applied Biosystems) using SYBR Green (Meissonnier et al., 2008). Each sample was analyzed in triplicate. Data were analyzed by a comparative CT method (Applied Biosystems). Transcript levels were calculated by normalizing to the levels of GAPDH mRNA. In addition, cytokines (IFN-γ, IL 4, TNF-α) in the spleen were analyzed in relation to the expression of antibodies (IgG, IgA) in the mucus, MLN and serum utilizing JMP software.^1^

### 2.6. ELISA for *Salmonella* and rSF

*Salmonella* Somatic or rSF ELISA was used to monitor antibodies in serum samples, lymphoid-associated tissue and intestinal mucosal from each piglet. For the determination of anti-rSF titers, 100 ng of purified rSF dissolved in 100 μl 0.1 M carbonate buffer (pH 9.6) was coated in each well of polystyrene 96-well flat-bottomed microtiter plates (Kangjia Ltd., China). For determining the anti-*Salmonella* Somatic antibody level, *S.* Choleraesuis C500 cells were diluted in PBS at 3 × 10^11^ CFU/ml and grown overnight. The cells were then harvested by centrifugation, inactivated for 10 min at 80°C and stored at −80°C. Plates coated with 100 μl of this suspension at 100-fold dilution in carbonate buffer were incubated at 37°C for 1 h, followed by overnight incubation at 4°C. Then they were blocked with a blocking buffer (PBS, 0.1% tween 20, and 5% skimmed milk). Samples of serum, lymphoid associated tissue and intestinal mucosa were diluted at the optimal dilution factor and added to each well and incubated at 37°C for 30 min. After three washes, plates were treated with biotinylated goat anti-pig IgG (Southern Biotechnology Inc., Birmingham, AL, USA) and lymphoid associated tissue homogenates or IgA at 37°C for 30 min, followed by five washes. The substrate solution TMB and H_2_O_2_ (50 μl) were then added to each well, and the plates were incubated at room temperature in the dark for approximately 10 min. The catalytic reactions were stopped with 50 μl 1% SDS. The optical densities were read at 630 nm using an ELISA reader (Liu, 2008).

### 2.7. Oral infection with *S.* Choleraesuis and STEC

The wild-type *S.* Choleraesuis strain C78-1 and the STEC field strain Ee were cultured in tryptose soy agar medium for 24 h at 37°C. The cultures were diluted of 1:100 in the tryptose soy broth and was incubated for 6 h with gentle shaking. All the pigs were fasted for 24 h prior to infection. The pigs were then intramuscularly injected with azaperone (stresnil, 4 mg/kg body weight), and 10 ml of a 10% (w/v) sodium bicarbonate solution was imported via gastric intubations followed by administration of bacterial emulsion. The Ee strain infection model was applied according to the method of B. T. Bosworth (Cornick et al., 1999). All of the pigs in this model were fed commercial rations containing 21.0% crude protein *ad libitum*, and until day 21 after the challenge, feed consumption, diarrhea, disorientation, abnormal behavior, and histopathology were all regularly assessed. For light microscopy, lungs and intestines were fixed in 10% formaldehyde solution embedded in paraffin and sections stained with Hematoxylin and eosin. Microscopically histological changes of these tissues caused by C78-1 and the Ee were the representative.

### 2.8. Statistical analysis

All data analysis was performed using the T Test or ANOVA in the SPSS 27 software^2^ for comparison of the differences in specific antibody levels between different groups. *P*-value less than 0.05 (typically < 0.05) was statistically significant.

## 3. Results

### 3.1. Preparation of recombinant *S.* Choleraesuis C520 (C500ΔasdΔcrp), C522 (C500ΔasdΔcrp vaccine expressing rSF), and C521 (C500ΔasdΔcrp vaccine expressing a control vector)

**FIGURE 1:**
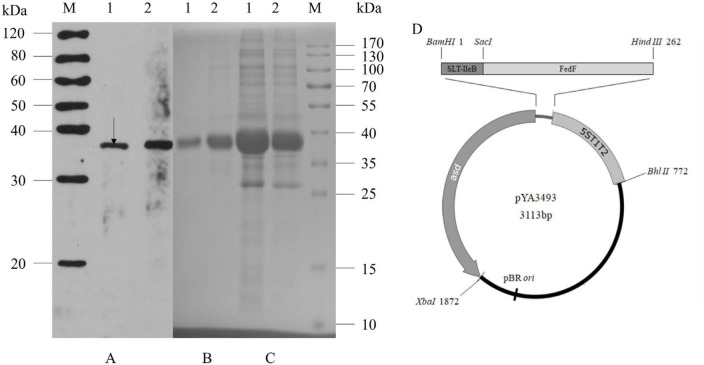
Expression of rSF by *S.* Choleraesuis C520, C522 and construction of vector pYA3493. C522 (pYA-SF; vaccine strain; rSF expression) and C521 (pYA3493; vector control) vaccine strains were cultured in LB broth at 37°C. Total cells (1.2 × 10^9^) and concentrated culture supernatants (750 μl at an OD600 of 0.8) were subjected to SDS-PAGE analysis, and rSF was detected by Coomassie blue staining or immunoblotting with anti-rSF rabbit polyclonal antibody. **(A)** Immunoblot of concentrated culture supernatants (lane 1) and total cell extracts (lane 2) of the C522 strain detected with anti-rSF rabbit polyclonal antibody. **(B)** Coomassie brilliant blue gel staining of concentrated culture supernatant from C522 (lane 1) and inclusion bodies from C522 (lane 2). **(C)** Coomassie brilliant blue-stained gel of total cell extracts from C522 (pYA-SF) (lane 1) and C521 (pYA3493) (lane 2). Molecular markers are indicated to the right. The position corresponding to the predicted MW of rSF protein is indicated by an arrow. **(D)** Construction of vector pYA3493.

To improve the *S.* Choleraesuis C500 vaccine, we introduced deletion mutations in both *asd* and *crp* genes. The resulting strain, C520, lacked the ability to synthesize DAP and was unable to grow on media without DAP, as expected. Its ability was restored when transfected with the control vector pYA3493, resulting in the creation of C521; or with the rSF expression vector pYA-SF, resulting in the creation of C522 (Figure 1D). The mean generation in Luria broth of the recombinant *S.* Choleraesuis strains C522 (31.2 min) and C521 (28.1 min) were similar to that of the parent attenuated C500 vaccine (27.9 min). We further compared their fermentation patterns on different carbohydrates. As expected, *S.* Choleraesuis C500, but not C521 and C522, was able to use maltose, glucose, mannose and xylose. Moreover, C521, C522 and the parent C500 strain were confirmed to share the same O and H antigenic type, 6, 7: C: 1, 5.

To confirm that *S.* Choleraesuis C522 expressed rSF we performed immunoblotting and Coomassie blue staining of SDS-polyacrylamide gels. C522 expressed the rSF of a molecular weight of 37 kDa, which is consistent with the calculated size of rSF (Figure 1A). Analysis of Coomassie blue-stained SDS-polyacrylamide gels showed that the amount of the rSF protein accounted for up to approximately 2.1% of the total C522 (pYA-SF) protein (Figures 1B, C). Approximately 69.8% of the rSF was detected in the cell lysates and 30.2% in the culture supernatants. To examine the stability of plasmids pYA3493 and pYA-SF in C522 and C521 *in vitro*, cells were cultured with daily passage of 1:1,000 dilution for five consecutive days in LB broth containing DAP. The last day, the amounts of the 37-kDa rSF that were expressed were similar to those from the first day, suggesting that the expression of rSF is stable from rearrangements.

### 3.2. The vaccine strains can colonize both the intestinal mucosa and lymphoid tissues

**FIGURE 2:**
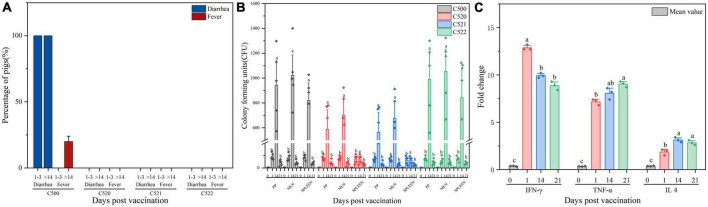
**(A)** Percentage of pigs experiencing diarrhea and fever 1–3 days and more than 14 days (>14) after vaccination C500, C520, C521, and C522. **(B)** Enumeration of the vaccine strains in lymphoid tissue after vaccination (day 0, day 1, day 14, and day 21). Pigs were orally vaccinated with 2.0 × 10^9^ CFU of C522 (pYA-SF) vaccine strain, C521 (pYA3493) vector control strain or C500 parental vaccine strain. Colonization of each vaccine strain from the Peyer’s patches (Facinelli et al., 2019). Facinelli et al. (2019), mesenteric lymph nodes (MLN) and spleen were measured on day 0, day 1, day 14, and day 21 post-vaccination. **(C)** Cytokine expression in the spleen from spleens of pigs immunized with C521. Pigs were orally vaccinated with 2.0 × 10^9^ CFU of C521 (pYA3493) vector control strain. Quantitative RT-PCR was performed to measure the level of interferon Y (IFN-Y), tumor necrosis factor-α (TNF-α) and interleukin 4 (IL 4) on day 0, day 1, day 14, and day 21 post-vaccination. Day 0 as a control group means that the pigs have been unvaccinated. The level of each cytokine gene was normalized to the corresponding GAPDH value. Data represent the means ± SE (*n* = 3). The alphabet indicate the statistically significant differences between CFU of Cytokine at 0, 1, 14, and 21 days post-vaccination. *P* < 0.05 was considered significant. Error bars indicate standard deviations.

To evaluate the colonization abilities of the engineered strains, we vaccinated swine. All C500-inoculated piglets had diarrhea from day 1 to day 3 post-vaccination, and 20% of C500-inoculated piglets had fever after 14 days post vaccination; however, piglets inoculated with C520, C522, or C521 did not show side effects such as diarrhea, fever, depressed spirit, or abnormal behavior during the corresponding period (Figure 2A). The vaccine strains were detected in rectal swabs collected from immunized piglets from day 1 to day 10 after oral immunizations, but only the C500 strain was recovered from shedding fecal samples from day 1 to day 3 post-vaccination. These findings suggest that the vaccine strains have the ability to colonize the intestine, which is vital to the mucosal immunity and also suggests that C521 and C522 are safer than their parent strain C500.

To verify that the vaccine strains can settle in lymphoid tissue and to assess the residence time, we monitored the quantities of vaccine strain in the Peyer’s patches, mesenteric lymph nodes and spleen at different times after inoculation. Between 0 and 1.4 × 10^3^ CFU were detected from day 0 to day 21 post-inoculation (Figure 2B). These results confirm that the vaccine strains can colonize both the intestinal mucosa and lymphoid tissues.

### 3.3. Vaccination with strain C521 induces proinflammatory cytokines

To further evaluate the cellular immune response to the C521 vaccine strain, we assessed the expression of inflammatory cytokines in the spleen of vaccinated piglets and naive piglets. On days 1, 14, and 21 after inoculation, the expression of IFN-γ, IL4, and TNF-α were elevated as assessed by qRT-PCR in the vaccinated piglets in contrast to piglets without inoculation of C521, on day 0. From day 1 to day 21 post-vaccination, IFN-γ expression level declined from day 1 to day 21 post-vaccination, whereas for TNF-α expression level increased. As for the expression of IL4, it sustains a certain level at three timepoints, day 1, 14, and 21 (Figure 2C). In addition, the correlation analysis showed that the Pearson correlation between the expression of cytokines (IFN-γ, IL 4, TNF-α) in the spleen and the expression of antibodies (IgG, IgA) in the mucus (Supplementary Table 1), MLN (Supplementary Table 2) and serum (Supplementary Table 3) was between 0.4 and 0.8, indicating better correlation. These results indicate that inoculation with strain C521 may elicit cellular immunity in swine.

### 3.4. The vaccine strains are potent at inducing an *S.* Choleraesuis-specific antibody response

**FIGURE 3:**
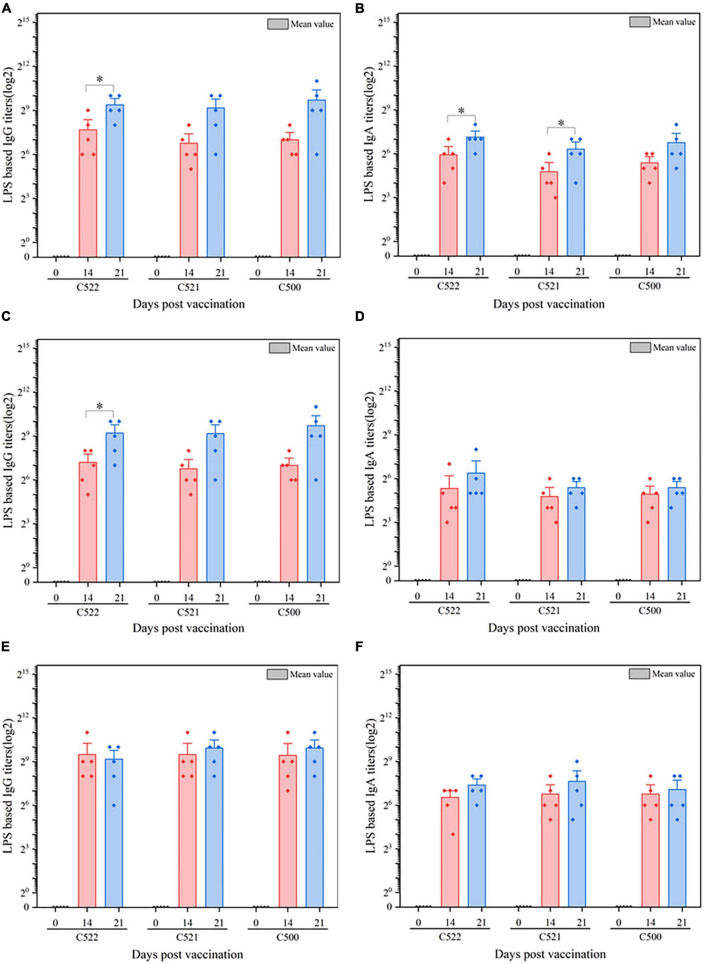
ELISA analysis of the anti-*S.* Choleraesuis immune response after oral vaccination of pigs with C521, C522 (pYA-SF) and C500-vaccinated pigs after oral vaccinations. Pigs were inoculated with the recombinant vaccine C522 (pYA-SF), C521 or parent (vector control) or the parental vaccine C500. Samples from 5 pigs were collected on day 0, day 14, and day 21. Individual pig serum, gut mucus (wash fluid from mucosa of terminal ileum) and MLN samples were tested for total IgG antibody or IgA antibody against whole *Salmonella* cells by ELISA. **(A,B)** Anti-*Salmonella* IgG or IgA titers obtained in serum; **(C,D)** anti-*Salmonella* IgG or IgA titers obtained in gut mucus; **(E,F)** anti-*Salmonella* IgG or IgA titers obtained in MLN. The data show the mean maximum end-point dilutions from the serum generating an optical density at 630 nm (OD630) two times that of undiluted pre-immune serum from the PBS-treated group (OD630 < 0.1). Statistical differences between groups were analyzed by the *T*-Test. The asterisk indicates the statistically significant differences (*P* < 0.05) between lipopolysaccharide (LPS) titers at 14 and 21 days post-vaccination. Error bars indicate standard deviations.

To further assess the immunogenicity of the vaccine strains, we evaluated the formation of antibodies against *S.* Choleraesuis in the sera, gut mucosa and mesenteric lymph nodes (MLN) of piglets that were orally vaccinated with C522, C521, and C500. ELISA was performed using whole cell *S.* Choleraesuis as antigen. C522, C521, and C500 vaccine elicited IgA and IgG antibody responses in the sera (Figures 3A, B), gut mucus (Figures 3C, D) and MLN (Figures 3E, F) on days 14 and 21 that were 7–9-fold higher than on day 0 (*P* < 0.01). Additionally, the antibody response to C522 was similar to that elicited by vaccination with C500 and C521. These results suggest C521 and C522 confer a vigorous systemic immune response, which verifies that the *asd* and *crp* deletions in these strains had a minimal influence on the immune response to *Salmonella* itself.

### 3.5. C522 elicits antibodies against rSF

**FIGURE 4:**
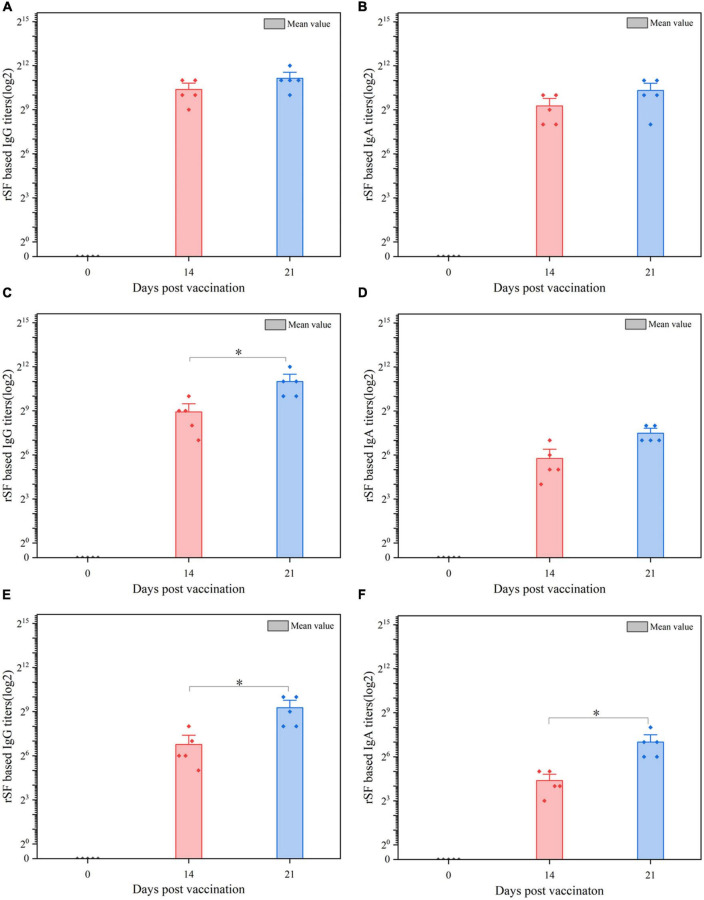
ELISA analysis of the anti-rSF immune response for pigs vaccinated orally with C522 (pYA-SF). Pigs were inoculated with the recombinant vaccine C522 (pYA-SF) on day 0, day 14, and day 21. Samples from 5 pigs in each group were collected at each time point. Individual pig serum, gut mucus and MLN samples were tested for total IgG antibody or IgA antibody against rSF by ELISA. **(A,B)** Anti-rSF IgG or IgA titers obtained in serum; **(C,D)** anti-rSF IgG or IgA titers obtained in gut mucus; **(E,F)** anti-rSF IgG or IgA titers obtained in MLN. The titers represent the maximum end-point dilutions from the sample yielding an optical density at 630 nm (OD630) two times that of undiluted pre-immune serum from the PBS-treated group (OD630 < 0.1). Mean values from each group were compared using the *T*-Test. The asterisk indicates the statistically significant differences (*P* < 0.05) between rSF titers at 14 and 21 days post-vaccination. Error bars indicate standard deviations.

We also assessed the production of antibodies against rSF by C522, C520, and C521, which is the only vaccine strain that expresses rSF. Our results demonstrate that C522-vaccinated pigs produced IgA and IgG antibody responses in the sera (Figures 4A, B), gut mucus (Figures 4C, D), and MLN (Figures 4E, F) at days 14 and 21.

### 3.6. Challenge of vaccinated pigs against *S.* Choleraesuis and STEC Ee

**FIGURE 5:**
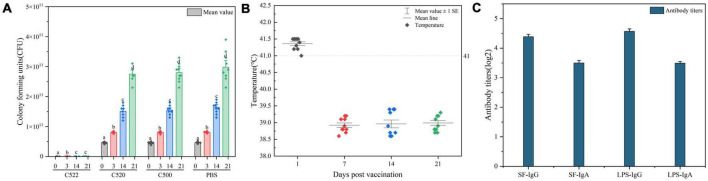
**(A)** CFU of Ee strain was detected in rectal swabs from pigs orally vaccinated with C522, C520, C500, and PBS from day 0 until day 21 post-challenge. The asterisk indicates the statistically significant differences between CFU of Ee strain at 0, 3, 14, and 21 days post-vaccination. *P* < 0.05 was considered significant. Error bars indicate standard deviations. **(B)** Temperatures of pigs during the 21 days post-vaccination. Pigs with temperatures above 41 degrees have an increase in body temperature. **(C)** ELISA analysis of the anti-rSF immune response in pigs vaccinated orally with C522 (pYA-SF) at 21 days after C78-1 and Ee challenge. Somatic of *S.* Choleraesuis C500-based IgG or IgA in mesenteric lymph nodes (MLN) from the C522-vaccinated pigs 21 days after C78-1 challenge and rSF-based IgG or IgA in MLN from the C522 vaccinated pigs 21 days after Ee challenge were determined by ELISA.

As a test of the efficacy of the vaccine strains, we assessed the ability of oral administration of the recombinant C521 vaccine, C522 vaccine, and the parental C500 vaccine to induce immunity in swine against oral challenge with an absolute lethal dose of the wild type, virulent parent strain, C78-1 (2 × 10^10^ CFU) or the STEC Ee strain (2.5 × 10^11^ CFU) (Zhao, 2009). Animals were fed a high-protein diet (comparable to commonly used commercial weaning rations). Complete protection against C78-1 was afforded by C521, C522, and C500, whereas there were no survivors in a group of ten naïve piglets in the PBS group (Table 3). Furthermore, almost no Ee strain was detected in rectal swabs from pigs orally vaccinated with C522 from day 3 until day 21 post-challenge, whereas large quantities were detected in the blank control group (Figure 5A). Only the C522 strain provided protection against STEC Ee. The other groups of animals showed clinical signs that were consistent with ED. Three pigs in the PBS group died of ED after seven days, and two were in lateral recumbence for 2 days and were euthanized. These results indicate that oral immunization with C521 or C522 (pYA-SF) can provide complete protection from *S.* Choleraesuis infection, and that C522 can additionally provide protection against STEC Ee.

**TABLE 3:** Effectiveness of oral immunization with the recombinant *S*. Choleraesuis C520 (pYA-SF) vaccine strain, the recombinant *S.* Choleraesuis C520 (pYA-3493), compared with the parental vaccine C500 and PBS, in protecting swine against challenge with wild-type parent C78-1 and STEC Ee.

Group	Immunizing strain	Immunizing dose (CFU)	Challenge dose (CFU)	Challenge strain	Survived pigs/total
A	C522	2.0 × 10^9^	2 × 10^10^	Ee	10/10
			2.5 × 10^11^	C78-1	10/10
B	C521	2.0 × 10^9^	2 × 10^10^	C78-1	10/10
			2.5 × 10^11^	Ee	0/10
C	C500	2.0 × 10^9^	2 × 10^10^	C78-1	10/10
			2.5 × 10^11^	Ee	0/10
D	PBS	200 μl	2 × 10^10^	C78-1	0/10
			2.5 × 10^11^	Ee	0/10

A: piglets were orally immunized once with the three vaccine strains or with PBS control and infected 3 weeks after the infections with wild-type *S. enterica* serovar Choleraesuis C78-1 and STEC Ee. Morbidity and mortality were observed and recorded daily for 21 days post-challenge. B: 2.0 × 10^10^CFU is representative of about 5 times the LD50 of C78-1 in non-immunized piglets. C: 2.5 × 10^11^ CFU is representative of about 5 times the LD50 of Ee in non-immunized piglets.

Continual fevers typically occur in pigs undergoing necrotic enteritis. To further determine the *in vivo* efficiency of the recombinant strains, we monitored the temperatures during the entire animal experiment. Vaccinated pigs had marginally increased in body temperature 1 day after inoculation, followed by temperatures that immediately return to basal levels (Figure 5B), which suggests that continued necrotic enteritis was not present.

**FIGURE 6:**
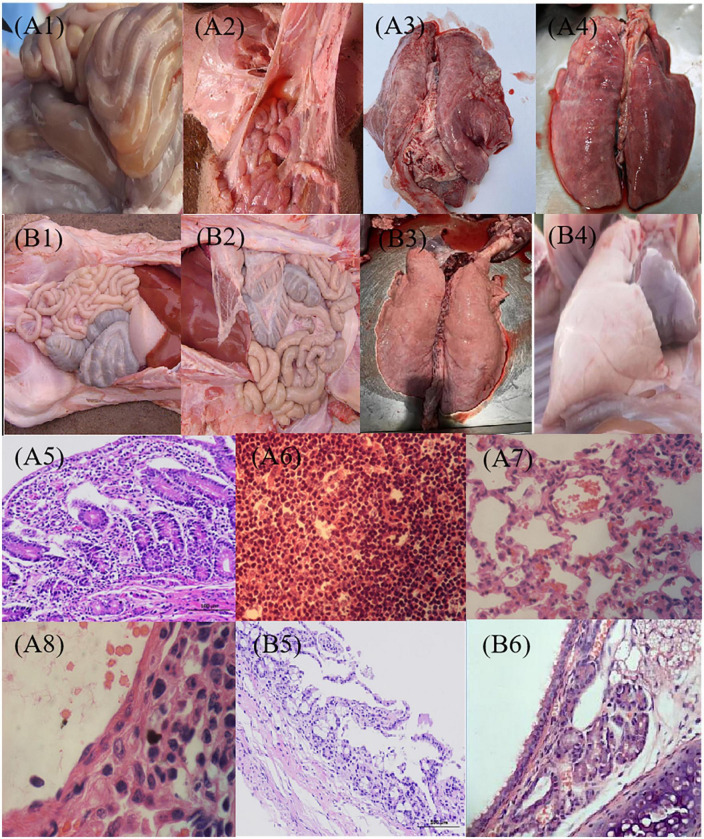
Pathological observation post-challenge with C78-1 and STEC Ee strains. Piglets were vaccinated orally with 1 dose of 2 × 10^9^ CFU of C522 live vaccine, or PBS as blank control. Three weeks later, the piglets were challenged orally with 2.0 × 10^10^CFU of virulent Salmonella C78-1 or 25 × 10^10^ CFU of STEC Ee field strain. **(A1,A2)** The PBS control of C78-1 and Ee on the gut, respectively. **(A1)** Mesenteric lymph nodes are diffusely hyperemic and mildly enlarged. **(A2)** There is moderate amount of yellowish fluid in the intestine, and the small intestine is hyperemic and bleeding. **(A3,A4)** The PBS control of C78-1 and Ee on the lung, respectively. **(A3)** Interstitial pneumonia, alveolar septal hyperemia, edema, inflammatory cell infiltration, septum widening, and alveolar shrinkage were seen in the lungs of piglets that challenged C78-1. **(A4)** The piglets that challenged Ee had fibrinous exudate on the apical lobes of their lungs, and the entire lung was covered with rubber-like exudate. **(A5)** The intestinal mucosal wall of piglets that challenged C78-1 is destroyed, the intestinal mucosa is detached, and inflammatory cells are infiltrated. **(A6)** The small intestinal lymph nodes of piglets that challenged Ee showed hemorrhagic and necrotic pathological manifestations. **(A7,A8)** The piglets that challenged C78-1 and Ee had hyperemia and thickened capillaries in the alveolar wall, and a large number of neutrophils and serous and fibrinous exudation in the alveolar cavity. **(B1,B2)** The protective effects of C78-1 and Ee on the gut after vaccination with C522, respectively. **(B3,B4)** The protective effects of C78-1 and Ee on the lung after vaccination with C522, respectively. **(B5)** The intestinal tissue of piglets in the C522 immune group had no lesions and the intestinal mucosa was intact. **(B6)** The lungs of piglets in the C522 immune group had no lesions. Magnification, 10 × 4 **(A5,B5)**, 10 × 40 (others).

We also directly examined tissues for pathologic signs of necrotic enteritis in 21 days after vaccination. Naive pigs (PBS) inoculated with C78-1 and Ee showed severe pathological changes in the lungs and intestines (Figures 6A1–8). All of these pigs had extensive systemic lesions including focal necrosis in the liver and lymph nodes, fibrous necrotic enteritis in the ileum and caecum and mild interstitial pneumonia. In contrast, no significant pathological lesions were observed for the vaccinated groups upon challenge with C78-1 and Ee (Figures 6B1–6). These findings confirm the efficacy of C522 in protection swine against both *S.* Choleraesuis and STEC Ee.

To verify the protective response in C522-vaccinated pigs, the IgG and IgA responses in the MLN were assessed on day 21. C522 induced high antibody (IgG and IgA) response against both rSF and *S.* Choleraesuis Somatic (Figure 5C). Similar results were observed in the sera and mucosa (Figure 4). These results demonstrate that rSF fusion protein expressed by the strain C522 confers systemic rSF-specific immunity.

## 4. Discussion

Attenuated *S.* Choleraesuis strains have been developed over the past decade as live vaccines for humans and animals to prevent diseases caused by *Salmonella* infection (Alborali et al., 2017). Recombinant *Salmonella* strains have also been developed as multivalent vaccines for delivering recombinant antigens that originate from viruses, bacteria and parasites (Atul et al., 2011). The advantage of mucosal delivery of these strains remain their ability to activate systemic as well as local and distant compartments of the immune system (DiGiandomenico et al., 2004). Additionally, to avoid safety problems associated with the usage of antibiotics for selection of expression vectors, a host-vector system called “balanced-lethal system,” based on the essential bacterial gene for aspartate β-semialdehyde dehydrogenase (*asd*), has been introduced to stabilize *Asd* + plasmids that carry foreign antigen genes (Santander et al., 2010). The *Asd* + plasmid pYA3493, which contains a DNA fragment encoding the β-lactamase signal sequence and 12 amino acid residues of the N terminus of mature β-lactamase from *Salmonella*, was designed and constructed for use in the periplasmic secretion of recombinant antigens for antigen delivery by *Salmonella* vaccines (Liang et al., 2008).

*S.* Choleraesuis C500, an attenuated vaccine strain attenuated by chemical methods, is highly immunogenic and relatively safe and has been used to prevent piglet paratyphoid in China for over 40 years (Zhao et al., 2009a). As yet, its mechanism of immune protection has been unclear (Ji et al., 2015). Moreover, it has residual toxicity, which limits its utility. A previous study demonstrated that the systemic immune response to C500 Δ*asd* is elicited via subcutaneous injection, but not via oral vaccination in mice (Zhao et al., 2009a). The primary reason for the reduced immunogenicity is that C500 Δ*asd* has a weakened colonizing ability compared to its parental C500 strain. On the other hand, the mechanism of antigen presentation of attenuated *S.* Choleraesuis is not always consistent in mice and in pigs (Dominguez-Bernal et al., 2008). In this study, we have shown that orally administered *Salmonella* C500 with both *asd* and *crp* deletions (C520) elicited a strong immune response and conferred protection against challenge with homologous strains in the natural host. C520 showed immunity that is similar to that of its parent strain C500 in pigs. Another recombinant strain, C522 (C500Δ*asd*Δ*crp*SF) was constructed to allow vector delivery of the rSF fragment, and our results show that it provided robust immunity either to STEC or to *Salmonella* itself. These findings support the use of C520 and its derivatives to confer mucosal and systemic immune response in swine (Burda et al., 2018).

To verify the efficacy of the strains that were derived from C500, we addressed their colonization ability in pigs. Large quantities of the vector control strain C521 invaded and colonized in intestinal mucosa, lymphatic related tissues (Peyer’s patches, MLN and spleen) at four time points post-vaccination. We also observed increased expression of IFN-γ, IL4 and TNF-α in spleens from pigs after oral vaccination of C521 and C500, suggesting that C521 may induce both humoral and cellular immunity in pigs. Because IL-4 can enhance CD8 T cell expansion during an immune response and IFN-γ can reciprocally counteract IL-4 induced reduction in IFN-γ production (Riber et al., 2015), the recombinant bacteria may induce a systemic immune response in pigs (Orndorff et al., 2000). Spleen levels of IL-4, TNF-α, and IFN-γ play a vital role in immune regulation, host defense against bacterial pathogens and protection from lethal bacterial infection (Ren et al., 2013). A simultaneous elevation of cytokines (IFN-γ and IL-4), which peaked at day 14 post-vaccination, may play a vital role in immune regulation, host defense against bacterial pathogens and protection at an early infection stage and thus complement the humoral immunity (Meissonnier et al., 2008; Ren et al., 2013). TNF-α level maintain a gently rising from day 1 to day 21 showing C522 can sustainedly attack host cell and live in the cell or colonize in the interstitial space, which contribute to the ability of triggering systemic immune response of C522. As expected, C521 conferred high antibody titers of *S.* Choleraesuis C500 Somatic IgA and IgG in intestinal mucosa, lymphoid associated tissues and sera. As a result, the vaccine provided complete protection efficiency against lethal challenge by C78-1.

A recombinant fusion gene, rSF, which is comprised of the B subunit of ST-IIe toxin fused to *fed*F adhesion of F18 fimbriae by a rigid linker, has previously been reported as an effective vaccine candidate (Liu, 2008). Therefore, in this study, we constructed a recombinant C520 strain using the C522 host strain that express rSF based on the *Asd* + balanced-lethal host-vector system.(Yan et al., 2013). As a model to further test the feasibility of C520-derived strains as mucosal vaccine vectors, we investigated the efficacy of C522 in preventing ED, a classical GI infectious disease caused by STEC that has a mortality rate of 90–100% and causes considerable economic loss in the swine industry (Hamabata et al., 2019). Although vaccines against F4 provide good protection from the PWD caused by F4 + ETEC, vaccines against F18 have not shown promising results due to insufficient immune response and difficulty producing specific antibodies (Okello et al., 2021). pYA-SF was stable (100% recovery) over 50 generations in the C522 vaccine strain grown in the presence of DAP. C520 contains pYA-SF expressed the rSF protein with an apparent molecular mass of about 37 kDa, and this protein was detected in the cytoplasm and in the culture supernatant. These results suggest that the signal peptide and 12 residues of the N terminus of β-lactamase (present in pYA-SF) promote periplasmic secretion of rSF. Kang et al. reported that the immunogenicity and appropriate sub-cellular localization of recombinant heterologously expressed antigen in a *Salmonella* vaccine strain augments immune responses by facilitating adequate exposure of rSF antigen to antigen-presenting cells for processing (Kang et al., 2002). Consistently, this study shows elevated protection efficacy against Ee strain and fecal excretion was also prevented when C522 was administered orally in pigs. Similar levels of anti-*S.* Choleraesuis IgG in the sera and IgA in the gut mucosa were induced by strains C500 and C522, suggesting that rSF-specific immunity, including mucosal and humoral immunity conferred by C522, did not interfere with immunity against *Salmonella* C520 itself. Additionally, the C522 strain elicited IgG and IgA that was specific to rSF. The results indicate that oral vaccination with this strain provides complete protection against challenge with Ee strain in a gastrointestinal tract model, suggesting that effective immunization might be achieved via the oral route based on this principle. Infection is initiated by the attachment of STEC organisms to intestinal brush border cells of the gastrointestinal tract; the bacteria then cause local damage to the gastrointestinal tract and systemic manifestations of disease (Cornick et al., 1999). Therefore, protection may correlate with the presence of protective antibodies in the intestinal mucosa, and induction of local immunity to this pathogen appears to be an ideal strategy for the prevention of infection (Melkebeek et al., 2013).

The efficacy of C522 in our study is in contrast to the results of another study in which a Δ*asd* strain harboring F1P2 could not provide sufficient immunity against *Bb* challenge via the oral inoculation route in mice (Han et al., 2014). Furthermore, only trace amounts of Ee strain was detected in rectal swabs from pigs orally vaccinated with C522 from day 3 until day 21 post-challenge, whereas copious quantities were detected in the blank control group. These findings suggest that protection correlates with the presence of specific IgG and IgA for rSF in the mucosa of the gastrointestinal tract, and protection against infection was apparently associated with the local systemic responses elicited by vaccination. In addition, these findings suggest that the degree of activation of gut-associated lymphoid tissue by oral vaccination is sufficient for antibody-secreting B cells to localize to the gastrointestinal tract lymphoid tissue (Liu, 2008). And these results also indicate that local lymphoid tissue is one of the sources of the protective antibodies. C522 conferred slightly higher IgA or IgG titers of the antibody against Somatic of *S.* Choleraesuis than did C520 in pigs, which verified that the rSF fragment delivery contributed to the elevated immunogenicity of the C520 vector.

## 5. Conclusion

C520 and the C522 antigen vector confer mucosal immune and systemic immune response in its natural host, swine. We showed that both C520 and C522 protect pigs against fatal infection with *S.* Choleraesuis. Furthermore, C522 provides a safe and promising vaccine candidate against STEC, which may be useful in practice in the future. This vaccine could also be easily adapted to develop multivalent recombinant *Salmonella* vaccines against other homologous strains.

## Data availability statement

The original contributions presented in this study are included in the article/Supplementary material, further inquiries can be directed to the corresponding authors.

## Ethics statement

The animal study was approved by the Animal Ethics Committee (AEC) of the Huazhong Agricultural University. The study was conducted in accordance with the local legislation and institutional requirements.

## Author contributions

GL, CL, and SL provided conceptualization, methodology, writing, reviewing, and editing. GL and SL provided methodology and investigation. CL and GL provided data analysis. AG provided methodology and visualization. BW provided project administration and manuscript checking. BW and HC provided supervision and visualization. All authors contributed to the manuscript and approved the submitted version.

## Footnotes

1
https://www.jmp.com/zhcn/software.html

2
https://www.ibm.com/docs/en/spss-statistics/27.0.0
