# Supplementary material for: C500 variants conveying complete mucosal immunity against fatal infections of pigs with Salmonella enterica serovar Choleraesuis C78-1 or F18+ Shiga toxin-producing Escherichia coli
Source: Front Microbiol. 2023 Sep 14;14:1210358. doi: 10.3389/fmicb.2023.1210358 (PMC10536267; doi:10.3389/fmicb.2023.1210358)
Supplement: Supplementary file 1 [file Table_1.DOCX]

**Table S1:** The Pearson correlation between the expression of pro-inflammatory cytokines in the spleen and the expression of antibodies in the mucus

|  | **IFN-γ** | **IL 4** | **TNF-α** | **IgG** | **IgA** |
| --- | --- | --- | --- | --- | --- |
| **IFN-γ** | 1.0000 | 0.97771 | 0.9912 | 0.4700 | 0.5472 |
| **IL 4** | 0.9771 | 1.0000 | 0.9694 | 0.6163 | 0.6820 |
| **TNF-α** | 0.9912 | 0.9694 | 1.0000 | 0.4826 | 0.5543 |
| **IgG** | 0.4700 | 0.6163 | 0.4826 | 1.0000 | 0.9924 |
| **IgA** | 0.5472 | 0.6820 | 0.5543 | 0.9924 | 1.0000 |

Note: The Pearson correlation coefficient of the expression of pro-inflammatory cytokines(IFN-γ, IL 4, TNF-α) in the spleen and the expression of antibodies (IgG, IgA) in the mucus was calculated between the residual obtained by linear regression of the expression of pro-inflammatory cytokines and days post vaccination and the residual obtained by linear regression of the expression of antibodies and days post vaccination. After adjusting for the effect of days post vaccination, the Pearson correlation between the expression of pro-inflammatory cytokines and the expression of antibody is between 0.4 and 0.8, indicating better correlation.

**Table S2:** The Pearson correlation between the expression of pro-inflammatory cytokines in the spleen and the expression of antibodies in the MLN

|  | **IFN-γ** | **IL 4** | **TNF-α** | **IgG** | **IgA** |
| --- | --- | --- | --- | --- | --- |
| **IFN-γ** | 1.0000 | 0.97771 | 0.9912 | 0.4182 | 0.6112 |
| **IL 4** | 0.9771 | 1.0000 | 0.9694 | 0.5829 | 0.7226 |
| **TNF-α** | 0.9912 | 0.9694 | 1.0000 | 0.4017 | 0.6396 |
| **IgG** | 0.4182 | 0.5829 | 0.4017 | 1.0000 | 0.6659 |
| **IgA** | 0.6112 | 0.7226 | 0.6393 | 0.6659 | 1.0000 |

Note: The Pearson correlation coefficient of the expression of pro-inflammatory cytokines(IFN-γ, IL 4, TNF-α) in the spleen and the expression of antibodies (IgG, IgA) in the MLN was calculated between the residual obtained by linear regression of the expression of pro-inflammatory cytokines and days post vaccination and the residual obtained by linear regression of the expression of antibodies and days post vaccination. After adjusting for the effect of days post vaccination, the Pearson correlation between the expression of pro-inflammatory cytokines and the expression of antibody is between 0.4 and 0.8, indicating better correlation.

**Table S3:** The Pearson correlation between the expression of pro-inflammatory cytokines in the spleen and the expression of antibodies in the serum

|  | **IFN-γ** | **IL 4** | **TNF-α** | **IgG** | **IgA** |
| --- | --- | --- | --- | --- | --- |
| **IFN-γ** | 1.0000 | 0.97771 | 0.9912 | 0.6579 | 0.4699 |
| **IL 4** | 0.9771 | 1.0000 | 0.9694 | 0.7598 | 0.5932 |
| **TNF-α** | 0.9912 | 0.9694 | 1.0000 | 0.6363 | 0.4918 |
| **IgG** | 0.6579 | 0.7598 | 0.6363 | 1.0000 | 0.5151 |
| **IgA** | 0.4699 | 0.5932 | 0.4918 | 0.5151 | 1.0000 |

Note: The Pearson correlation coefficient of the expression of pro-inflammatory cytokines(IFN-γ, IL 4, TNF-α) in the spleen and the expression of antibodies (IgG, IgA) in the serum was calculated between the residual obtained by linear regression of the expression of pro-inflammatory cytokines and days post vaccination and the residual obtained by linear regression of the expression of antibodies and days post vaccination. After adjusting for the effect of days post vaccination, the Pearson correlation between the expression of pro-inflammatory cytokines and the expression of antibody is between 0.4 and 0.8, indicating better correlation.
